# Thalamic Hemiplegia

**Published:** 1907-11-16

**Authors:** Purves Stewart


### Neurology.

### THALAMIC HEMIPLEGIA.

By PURVES STEWART, M.D., F.R.C.P.

Within the past few years considerable attention has been devoted to a more minute analysis of the various forms of hemiplegia. To Dejerine and Roussy belongs the credit of having, as recently as last year, established on a secure anatomical basis a striking symptom-complex which is characteristic of a lesion localised to the postero-external part of the optic thalamus. It has for some time been recognised that the thalamus is the most important sub-cortical station through which sensory impressions are transmitted to the cortex, mainly, to the post-central convolution. Lesions strictly limited to the thalamus are somewhat uncommon, and perhaps the best idea of the clinical syndrome will be obtained by recounting a case which I recently had the opportunity of observing.

A male patient (now 51 years of age), seven years ago, after a period of severe domestic worry, was suddenly seized with hemiplegia affecting the right face, arm, and leg. There was no unconsciousness, no aphasia, no hemianopia. The hemiplegic motor weakness cleared up within a few weeks, and he can now perform voluntary movements at all joints. The deep reflexes in the affected limbs are, if anything, slightly increased, but the plantar reflex remains of the normal flexor type, indicating that the lesion has not interrupted the pyramidal motor path. Ever since the onset, however, there has been marked diminution of sensation to all forms of stimulation of the affected side, consisting of cutaneous anæsthesia and analgesia of moderate intensity, most marked at the periphery of the limbs and diminishing as we approach the median plane. To cold and heat, however, the affected area is unduly sensitive. There is also marked impairment of joint-sense and of osseous sensation as tested by means of a vibrating tuning-fork. Together with this there is astereognosis of the affected hand, *i.e.* inability to recognise objects by feeling them without the aid of vision. In addition there have gradually developed spontaneous irregular movements of the affected limbs of a choreiform or athetoid variety, and there is marked hemi-ataxy on voluntary movement. But what is more characteristic of all, the patient complains of paroxysms of spontaneous pain, of a burning or stabbing character, in the face and limbs of the affected side of the body. These pains are intolerable in their severity, and have resisted all the analgesic remedies that have yet been tried.

Such are the characters of a typical case of thalamic lesion. In it we notice that whilst the initial motor paralysis cleared up rapidly, the sensory impairment and the spontaneous pains have, on the contrary, remained; in fact, the pains have become progressively worse. Such dissociation of sensory and motor phenomena can only be produced by a lesion at some part of the sensory tract where it is not intersected by the pyramidal motor path Neither in the cortex nor in the internal capsule is this possible, and as a matter of fact Dejerine and Roussy have demonstrated anatomically in several similar cases that the lesion is in the posterior and external part of the optic thalamus. Somewhat similar clinical phenomena in the way of sensory impairment and hemi-ataxy may be produced by lesions in the corpora quadrigemina, but in the latter case there are no spontaneous pains; moreover quadrigeminal lesions are usually associated with ocular paralyses of a supranuclear type.

